# A study of quantitative indicators for slice sorting in cine-mode 4DCT

**DOI:** 10.1371/journal.pone.0272639

**Published:** 2022-08-26

**Authors:** Changhwan Kim, Hojae Kim, Sung-woo Kim, Youngmoon Goh, Min-jae Park, Hojin Kim, Chiyoung Jeong, Byungchul Cho, Eun Kyung Choi, Sang-wook Lee, Sang Min Yoon, Su Ssan Kim, Jin-hong Park, Jinhong Jung, Si Yeol Song, Jungwon Kwak

**Affiliations:** 1 Department of Radiation Oncology, Yonsei Cancer Center, Yonsei University College of Medicine, Seoul, Republic of Korea; 2 Department of Radiation Oncology, Yonsei Cancer Center, Seoul, Republic of Korea; 3 Department of Radiation Oncology, Asan Medical Center, Seoul, Republic of Korea; 4 Department of Radiation Oncology, Asan Medical Center, University of Ulsan College of Medicine, Seoul, Republic of Korea; University of the Pacific Arthur A Dugoni School of Dentistry, UNITED STATES

## Abstract

The uncertainties of four-dimensional computed tomography (4DCT), also called as residual motion artefacts (RMA), induced from irregular respiratory patterns can degrade the quality of overall radiotherapy. This study aims to quantify and reduce those uncertainties. A comparative study on quantitative indicators for RMA was performed, and based on this, we proposed a new 4DCT sorting method that is applicable without disrupting the current clinical workflow. In addition to the default phase sorting strategy, both additional amplitude information from external surrogates and the quantitative metric for RMA, investigated in this study, were introduced. The comparison of quantitative indicators and the performance of the proposed sorting method were evaluated via 10 cases of breath-hold (BH) CT and 30 cases of 4DCT. It was confirmed that N-RMSD (normalised root-mean-square-deviation) was best matched to the visual standards of our institute’s regime, manual sorting method, and could accurately represent RMA. The performance of the proposed method to reduce 4DCT uncertainties was improved by about 18.8% in the averaged value of N-RMSD compared to the default phase sorting method. To the best of our knowledge, this is the first study that evaluates RMA indicators using both BHCT and 4DCT with visual-criteria-based manual sorting and proposes an improved 4DCT sorting strategy based on them.

## 1. Introduction

Four-dimensional computed tomography (4DCT) has been widely utilised to overcome the adverse influences of significant tumour movement due to a patient’s respiration on radiotherapy procedures [1–13]. 4DCT reduces motion artefacts and resultant systematic uncertainties in CT simulation and the subsequent planning of radiation treatment [14–17]. Cine-mode scan, one of the mainly employed 4DCT scan protocols, allows acquired time-series CT slices to be sorted according to the respiratory information after CT reconstruction.

Surrogate respiratory signals extracted from external devices, including reflective markers and elastic belts, are generally used to index and match the respiratory information on 4DCT. After the acquisition during CT scans, surrogate respiratory signals can be labelled on oversampled CT data acquired at each couch position and utilised for 4DCT sorting. It is mainly performed based on phase information calculated by equally dividing each cycle of the patient’s surrogate respiratory signals. The image segments closest in time to each breathing phase at each couch position are grouped by selecting CT images with the lowest phase error at each phase bin.

**Fig 1:**
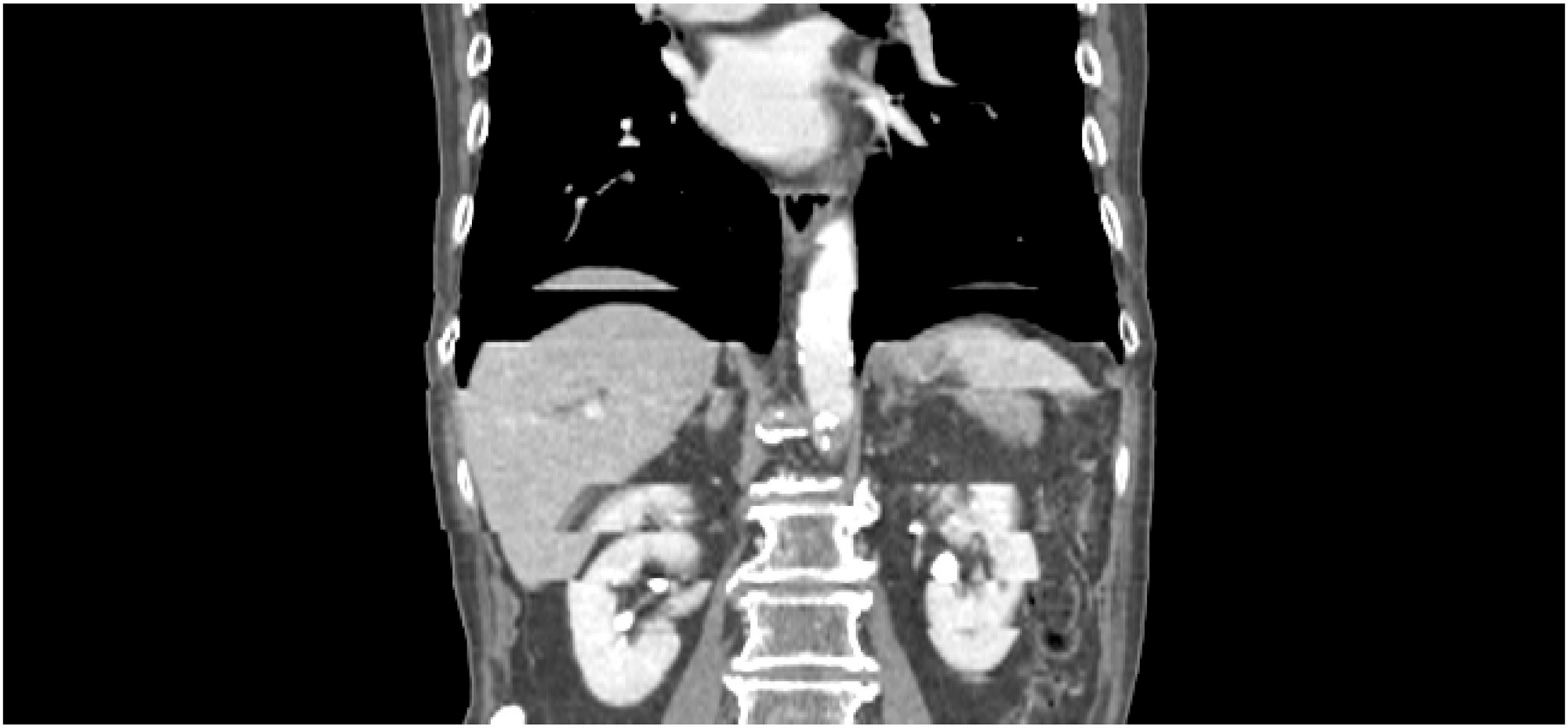
Illustration of RMA in coronal view of cine-mode 4DCT.

However, phase sorting of cine-mode 4DCT has several vulnerable points. Because the projection data for CT reconstruction were not obtained simultaneously, each CT slice obtained with cine mode contains inherent motion artefacts. Additionally, due to the characteristics of the cine mode, image artefacts with discontinuous band shape between couch positions can occur in coronal and sagittal views of 4DCT, as shown in Fig 1. Moreover, if the patient’s respiration is irregular and non-reproducible, or the surrogate signals obtained from external devices have a low correlation with the patient’s actual internal motion, the respiratory information conjugated with CT data might be inaccurate [18–20]. Therefore, motion artefacts frequently appear in 4DCT images despite 4DCT sorting techniques being applied. Those motion artefacts induced by the irregular respiratory patterns and low correlation of the surrogate respiratory signal with internal motion are called as 4DCT uncertainties, or residual motion artefacts (RMA).

For RMA reduction to minimise the degradation of the quality of overall radiotherapy, various methods have been attempted [21–29]. One of the methods for clinical applications is the image similarity-based method between CT images from adjacent couch positions [30–32]. This method utilises the similarity of adjacency as the similarity between CT images acquired at the same respiratory phase bin would be high, in general. Therefore, it is usually employed as a quantitative indicator for RMA, formulated in various ways. An index called normalised cross-correlation (NCC) is commonly utilised among them. This image similarity-based method is the most feasible and practical for clinical applications among various 4DCT sorting methods, but there are still several points to be improved. First, those quantitative indicators based on the similarity of adjacent CT images are calculated from the pixel values only; therefore, it may not reflect the relationship between pixels having the same value at different positions. Further, the CT images sorted by image similarity may be misguided to the suboptimal condition far from the patient’s actual respiration, even though resultant images have no discontinuities. As 4DCT acquired with the cine mode would contain intrinsic RMA, CT images with some degree of image discontinuity may be closer to the patient’s actual respiration. Besides, the similarity index values can be high despite many uncertainties in the CT images in reality. For instance, if the amplitude of respiration is uneven during the 4DCT scan, positional inversion on CT images along the Z-direction can occur. As a result, the image at the nth couch position becomes similar to the image at the n+1th couch position, or vice versa. In this case, the similarity value of two adjacent images will be high and thus, the similarity-based sorting method may mislead to worse RMA contamination.

As there is no complete solution to date that accounts for all these weaknesses and can be applied in every clinical practice, our institute conducts the manual sorting method for 4DCT. The conventional phase sorting method is firstly applied using the surrogate respiratory signal; our hospital utilises an RPM (real-time position management) system (Varian Medical Systems, Palo Alto, CA). Then, based on the visual inspection of an experienced operator, the CT slices with severe RMA are manually filtered out and replaced with the most plausible alternative image within the similar respiratory phase level. Although it may be challenging to maintain consistent manual sorting results due to intra- and interobserver variabilities from the subjective standards of the operators, it is believed to be the most straightforward and effective method in clinical practice. However, as the CT images of each phase in all couch positions should be monitored, a long and laborious process is inevitable; it usually takes about 20 to 30 minutes to sort one dataset of 4DCT images manually.

Therefore, we propose a new method that can replace the manual sorting method and compensate for the shortcomings of the existing sorting methods. Specifically, this study aims to minimise RMA, caused by both irregular respiration and the low correlation between surrogate respiratory signals and actual internal motions of the patient, while maintaining the phase sorting based 4DCT workflow. To attain optimal image quality of the sorted 4DCT without significant distortions of the actual respiration, we propose a new sorting strategy that introduces two additional information to the phase sorting: amplitude information and quantitative indicator for RMA. From the RPM respiratory signals of the patient, the representative respiratory signal of the patient is modelled, and the resulting model can be regarded as a reference signal. Then, the amplitude error can be calculated, and introducing it into the sorting procedure could reduce 4DCT uncertainties, especially in irregular respirations.

A similarity-based 4DCT uncertainty index can further reduce the RMA, mainly caused by the low correlation between the RPM respiratory signals and the actual motions of internal organs. However, as described above, because some drawbacks may arise when image sorting is performed based on the existing similarity-based metrics, a comparative study was conducted first to evaluate various types of RMA indicators and select one that best represents the amount of 4DCT uncertainties among them. For the studies, breath-hold (BH) CT as an artefact-free reference data and 4DCT and the corresponding uncertainty scores visually evaluated by the operators of our institute were utilised.

Through the comparative study, a quantitative index that conforms with the qualitative basis of manual sorting was selected to be reliably employed as a criterion for image sorting in 4DCT. Thus, the sorting method of 4DCT combining the phase, amplitude, and similarity-based 4DCT uncertainty index was suggested in this work. This study was approved by the Institutional Review Board (IRB) of Asan Medical Center (approval number: S2020-3105-0001). All data were fully anonymized before investigators accessed them. Given the retrospective nature of the study, written informed consent was waived by the IRB. Employing the retrospective studies of patients’ 4DCT data with the approval of the IRB, we validated that the proposed method can effectively sort 4DCT images and reduce RMA. To the best of our knowledge, this is the first study that evaluates 4DCT uncertainty indicators using both BHCT and 4DCT with visual-criteria-based manual sorting, and proposes an improved 4DCT sorting strategy based on them.

## 2. Methods

### 2.1. Comparative studies of quantitative indices for RMA

#### 2.1.1. Candidates of quantitative indices for RMA

We conducted a comparative study of three types of quantitative indicators for 4DCT uncertainties, based on the principle of quantifying the uncertainties from the similarity between adjacent CT images.

The first type of quantitative index used in this study is NCC. NCC can be calculated according to the following equation from the pixel values of two CT images of adjacent couch positions.

$$NCC\left(I_{N},I_{N+1}\right)=\frac{\sum_{x,y}\left[I_{N}\left(x,y\right)-{\overset{-}{I}}_{N}\right]\left[I_{N+1}\left(x,y\right)-{\overset{-}{I}}_{N+1}\right]}{\sqrt{\sum_{x,y}{\left[I_{N}\left(x,y\right)-{\overset{-}{I}}_{N}\right]}^{2}{\left[I_{N+1}\left(x,y\right)-{\overset{-}{I}}_{N+1}\right]}^{2}}},$$

*I*_*N*_ (*x*, *y*) and *I*_*N*+1_ (*x*, *y*) are the pixel values of *I*_*N*_ and *I*_*N*+1_, which are images at the N^th^ and N+1^th^ couch position, respectively. ${\overset{-}{I}}_{N}$ and ${\overset{-}{I}}_{N+1}$ are the mean pixel values of images *I*_*N*_ and *I*_*N*+1_. NCC can have a value from 0 to 1. It will be close to 1 if the respiratory information of the two CT images of the adjacent couch positions is well matched, equivalent to having fewer RMA in the CT images.

The next type of quantitative index is a root-mean-square deviation (RMSD). Principally similar to NCC, RMSD is defined as the square root of the mean squared error, and it can be expressed as shown below, where *K* is the number of pixels in the images *I*_*N*_ (*x*, *y*) and *I*_*N*+1_ (*x*, *y*). In contrast to NCC, RMSD will be close to zero if there are fewer 4DCT uncertainties, whereas having many uncertainties will result in larger values.


$$RMSD\left(I_{N},I_{N+1}\right)=\frac{\sqrt{\sum_{x,y}{\left[I_{N}\left(x,y\right)-I_{N+1}\left(x,y\right)\right]}^{2}}}{K}$$

**Fig 2:**
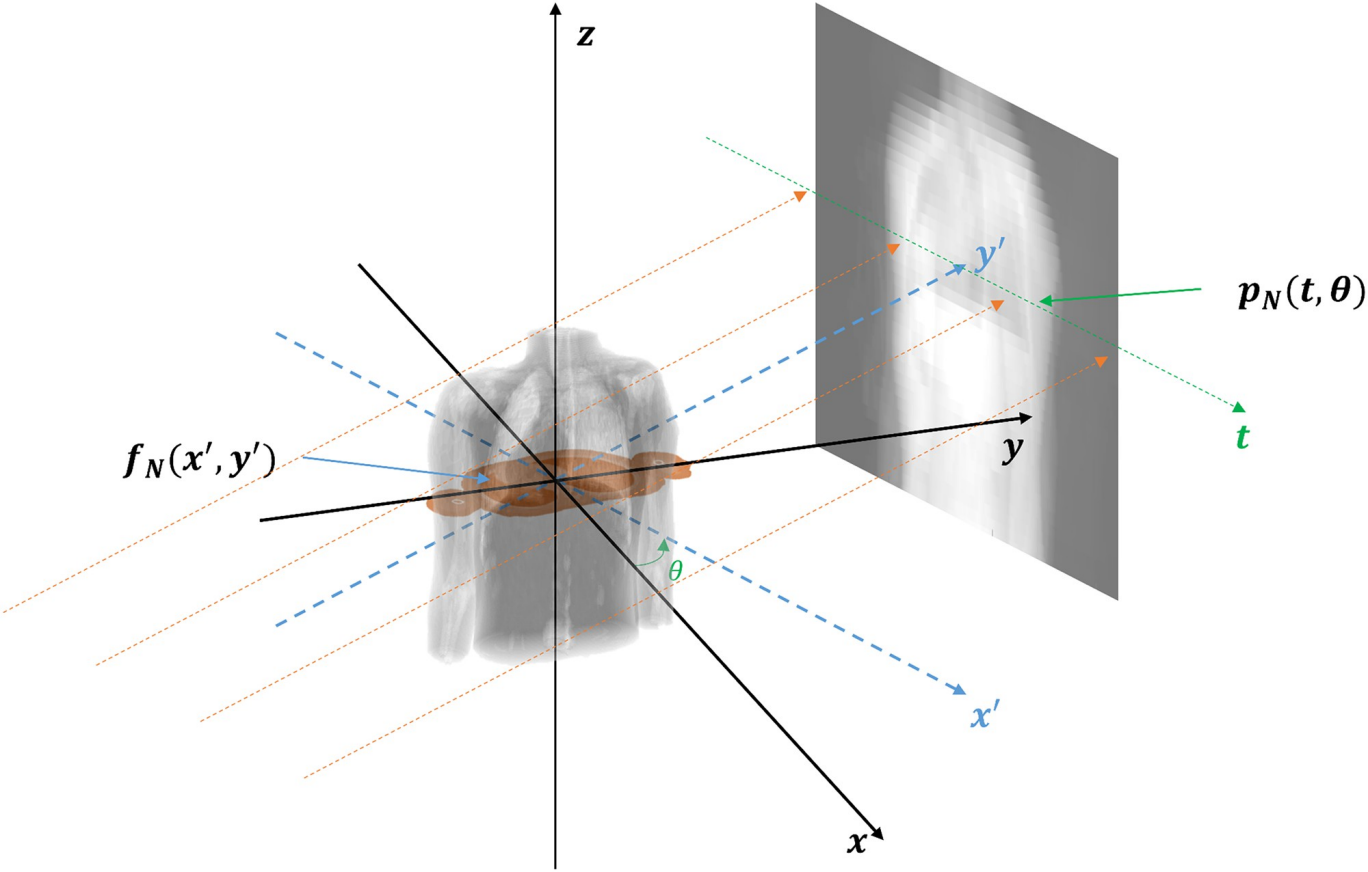
Rotated coordinate system in parallel-beam geometry.

The next indicator is based on the data consistency condition (DCC). DCC will depend mainly on the RMA, with the assumption that other factors such as scatter, and suboptimal calibration are managed within a tolerable level [33–36]. Therefore, the amount of 4DCT uncertainties can be quantitatively expressed by employing the consistency index of DCC as described below. Based on this concept, the consistency index of two CT images of adjacent couch positions would be close to 1 if there are fewer RMA.

$$D_{N}=\int p_{N}\left(t,\theta \right)dt=\iint f_{N}\left(x^{\prime },y^{\prime }\right)dx^{\prime }dy^{\prime }$$

(*forward projection in parallel beam geometry*,*as displayed in* Fig 2)

$$DCC(I_{N},I_{N+1})=\frac{\text{min}\left(D_{N},D_{N+1}\right)}{max(D_{N},D_{N+1})}$$

**Fig 3:**
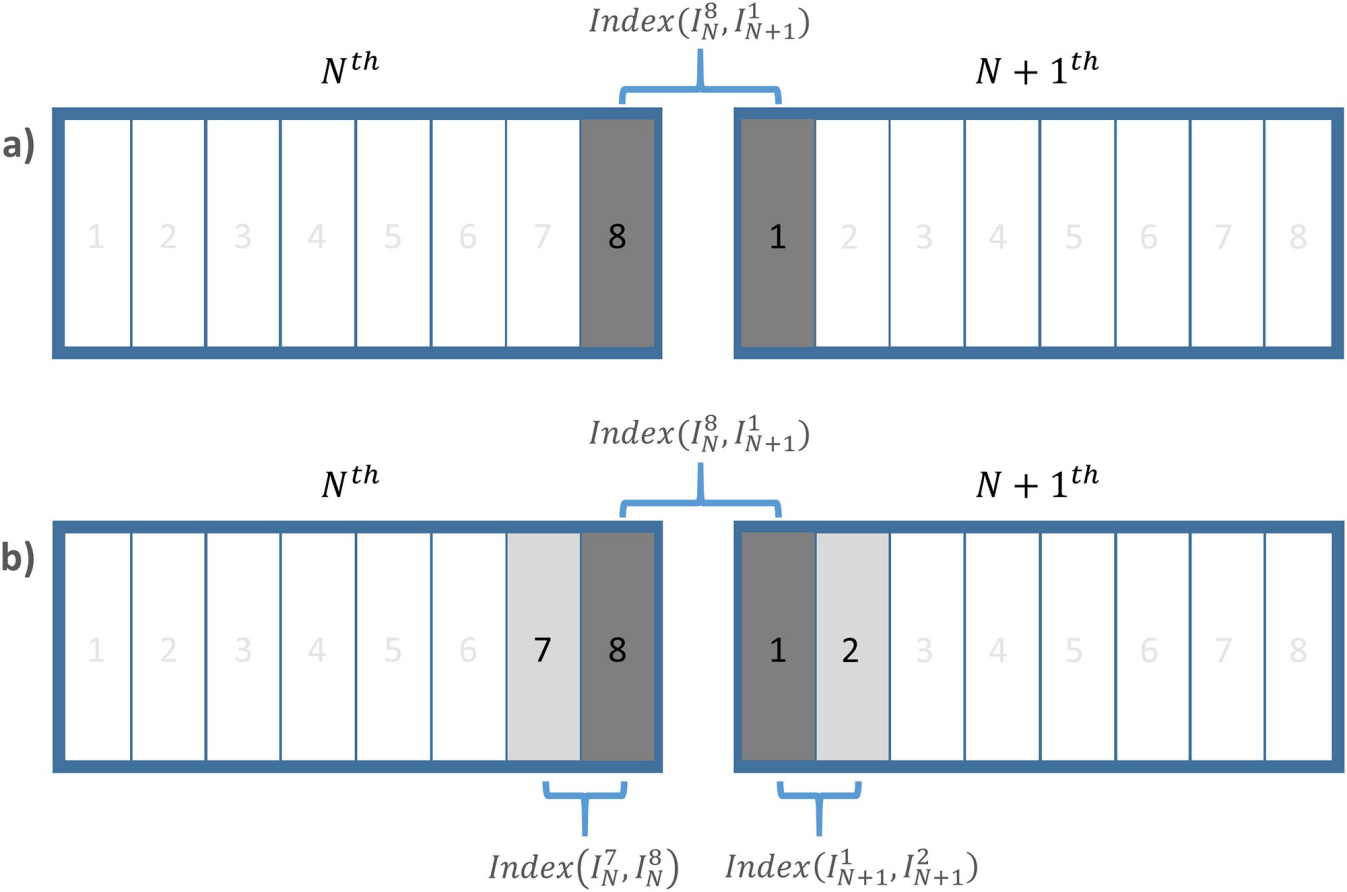
Schematic of the quantitative index for RMA based on the similarity between adjacent CT slices. (a) The quantitative index without the normalisation process and (b) with the additional normalisation process.

Moreover, indicators in conjunction with the additional normalisation procedure were also introduced to streamline the distribution of their values regardless of the scanned body position. The indices with the normalisation procedure can be additionally governed using the neighbouring image, as shown in Fig 3. After the pre-acquisition of quantitative values between the two N^th^ images and the two N+1^th^ images, the calculation can be conducted once more with the two primary results to perform the normalisation. N-NCC, N-RMSD, and N-DCC are quantitative indices introducing the above normalisation procedure to NCC, RMSD, and DCC, respectively. In summary, a comparative analysis was performed on six quantitative indicator candidates. All indices were calculated only within the patient body region of CT data.


$$Normalised\;Index=\frac{Index(I_{N}^{8},I_{N+1}^{1})}{mean(Index\left(I_{N}^{7},I_{N}^{8}\right),Index\left(I_{N+1}^{1},I_{N+1}^{2}\right))}$$

#### 2.1.2. Verification of quantitative indices using breath-hold CT

**Fig 4:**
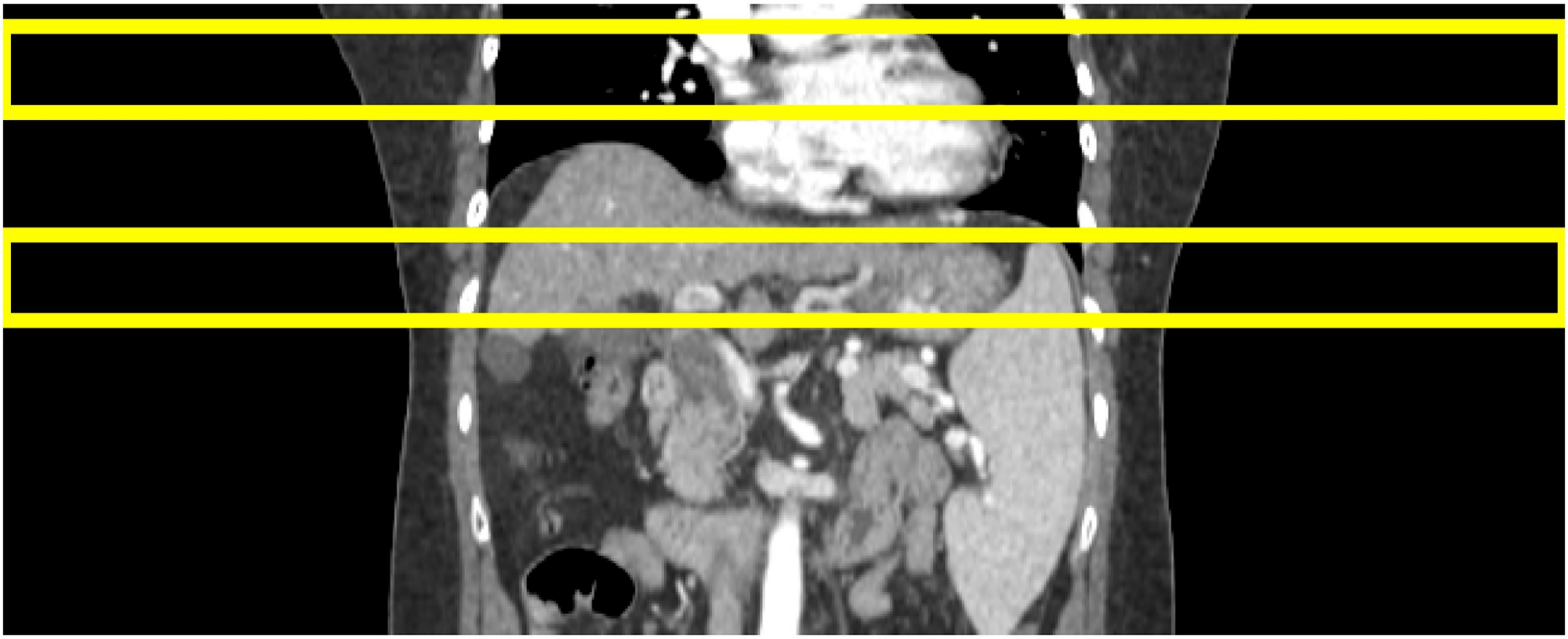
Evaluation regions of quantitative indicators for RMA in coronal view of BHCT. Top: Thoracic region; Bottom: Abdominal region.

We first checked that those indicator candidates were appropriately formulated to represent the amount of RMA regardless of the body parts in 4DCT uncertainty-free situations. By checking whether the index candidates on BHCT show a value close to the ideal extreme value, such as 0 or 1, one can validate that there is no abnormality in the fundamental design of each indicator. Then, each quantitative index was calculated by dividing the evaluation region into thoracic and abdominal regions, as shown in Fig 4.

#### 2.1.3. Correlation of the quantitative index with the operator’s qualitative standard

Next, we conducted another comparative study to check how much each quantitative metric coincides with the visual criteria of workers performing 4DCT manual sorting tasks in our institute. If each quantitative indicator is highly correlated with the visual criteria of the workers, it can be said that the corresponding index can well represent the amount of RMA. Therefore, it can be sensibly employed as a criterion for 4DCT sorting.

**Fig 5:**
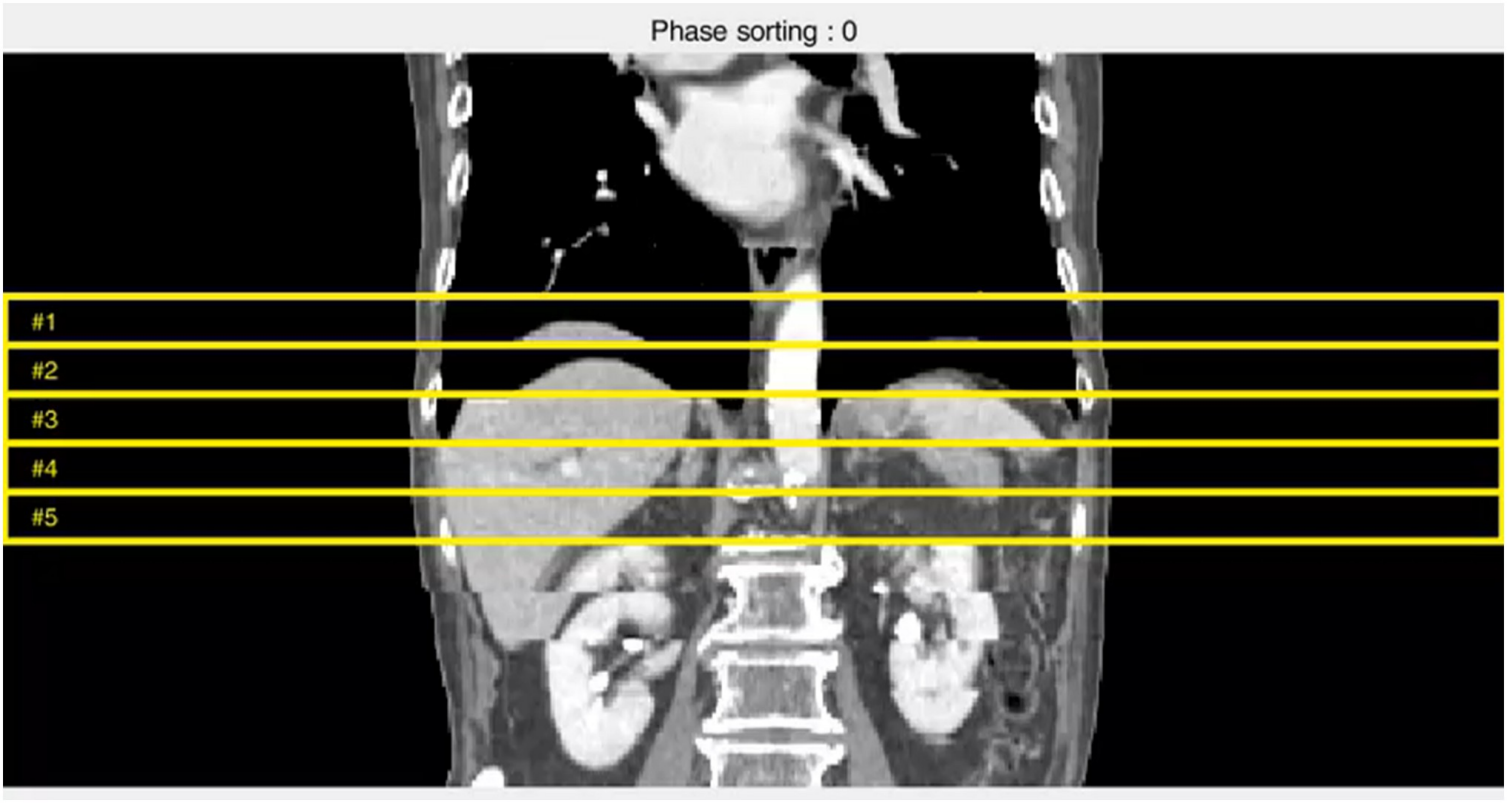
Coronal view of phase-sorted 4DCT and its evaluation regions of quantitative indicators for RMA.

First, the following evaluation procedure was performed to quantify the visual standards of workers. For the phase sorted 4DCT data set, five sections near the CT isocentre were designated as the region of evaluation (Fig 5). For each section, two operators respectively scored from 1 to 4 points according to the amount of RMA, described in Table 1. One point was given to the case with the least RMA, and four points were assigned in the case of the most severe uncertainties. If it is determined that manual sorting is necessary, three or more points were assigned to provide additional criteria for the middle range of the point. After scoring, the inter-rate agreement of the scores of two workers was evaluated to confirm the consistency of the visual criteria of the workers through weighted kappa analysis. Also, each quantitative indicator for RMA was calculated for each slice, and we analysed the degree of correlation between the evaluated scores and those metrics by using Pearson correlation analysis.

**Table 1:** Scoring of the amount of RMA of 4DCT.

	Degree of RMA
Manual re-sorting not required	1
	2
Manual re-sorting required	3
	4

### 2.2. Proposed method: Amplitude-weighted phase sorting method with image similarity (Amp-sim-w)

#### 2.2.1. Concept

As mentioned in the introduction section, 4DCT uncertainties can be largely categorised into two: uncertainties caused by irregular and non-reproducible respirations, and uncertainties caused by low correlation between surrogate respiratory signals and internal motions of organs.

For 4DCT uncertainties due to irregular respirations, the phase sorting method is somewhat resistant to inaccurate phase information. Phase error is the difference between the actual acquired phase and the certain reconstructed breathing phase level. As phase sorting selects CT images with the minimal phase error, this can reasonably prevent images of different respiratory phases from being classified into the same respiratory phase bin. Further, the scheme of phase reallocation by equally dividing every peak-to-peak of surrogate respiratory signals can additionally restrain phase misalignments due to irregular respirations. Meanwhile, since the phase sorting-based workflow assumes that the same respiratory phase level would have the same amplitude level, there is essentially no way to consider amplitude misalignment. Therefore, amplitude and phase nonalignments should be considered to reduce 4DCT uncertainties due to irregular and non-reproducible respirations.

In the case of the low correlation between surrogate respiratory signal and internal motion of the patient, severe RMA might be inevitable despite CT images with similar surrogate respiratory information including phase and amplitude being well sorted. Therefore, it is best to further use information from the acquired CT images in this situation. One such method is to use the image similarity-based 4DCT uncertainty indicator. By applying a proper RMA index, it is possible to reinforce insufficient information due to the low correlation issue, thereby allowing the reduction of 4DCT uncertainties.

Therefore, we proposed the amplitude-weighted phase sorting method with image similarity (Amp-sim-w), which introduces not only the respiratory phase information but also the amplitude information and a quantitative indicator as an objective function of 4DCT sorting. As a result, it is believed that the RMA due to both irregular respiration and low correlation can be considerably reduced. To sum up, using the current phase sorting framework, the proposed method can improve the quality of resultant images of 4DCT sorting without artificial distortions of the respiratory signal, additional device and computational burden. The detailed workflow of the proposed method is described in the following subsection.

#### 2.2.2. Workflow

**Fig 6:**
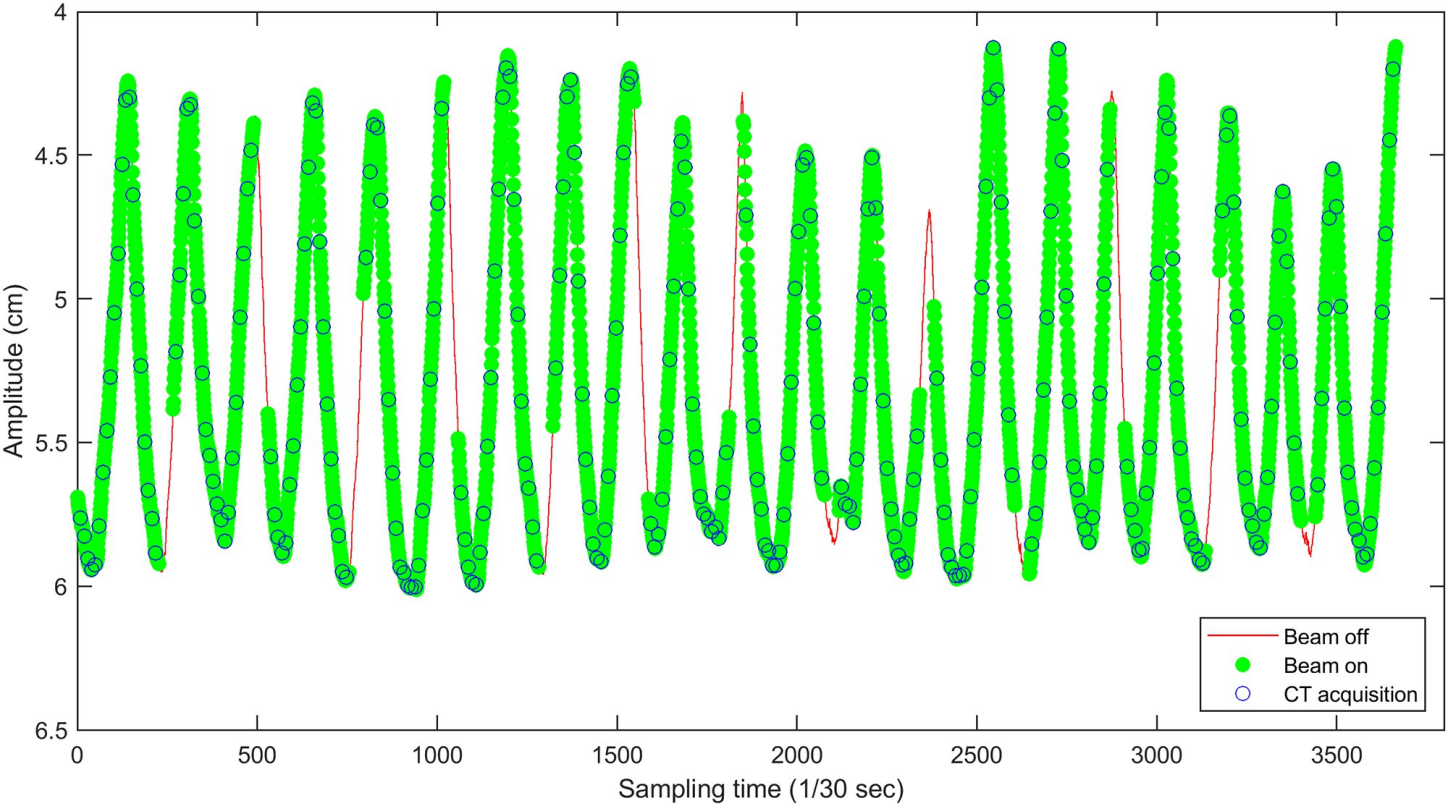
Surrogate respiratory signal obtained by the RPM system during a 4DCT scan.

**Fig 7:**
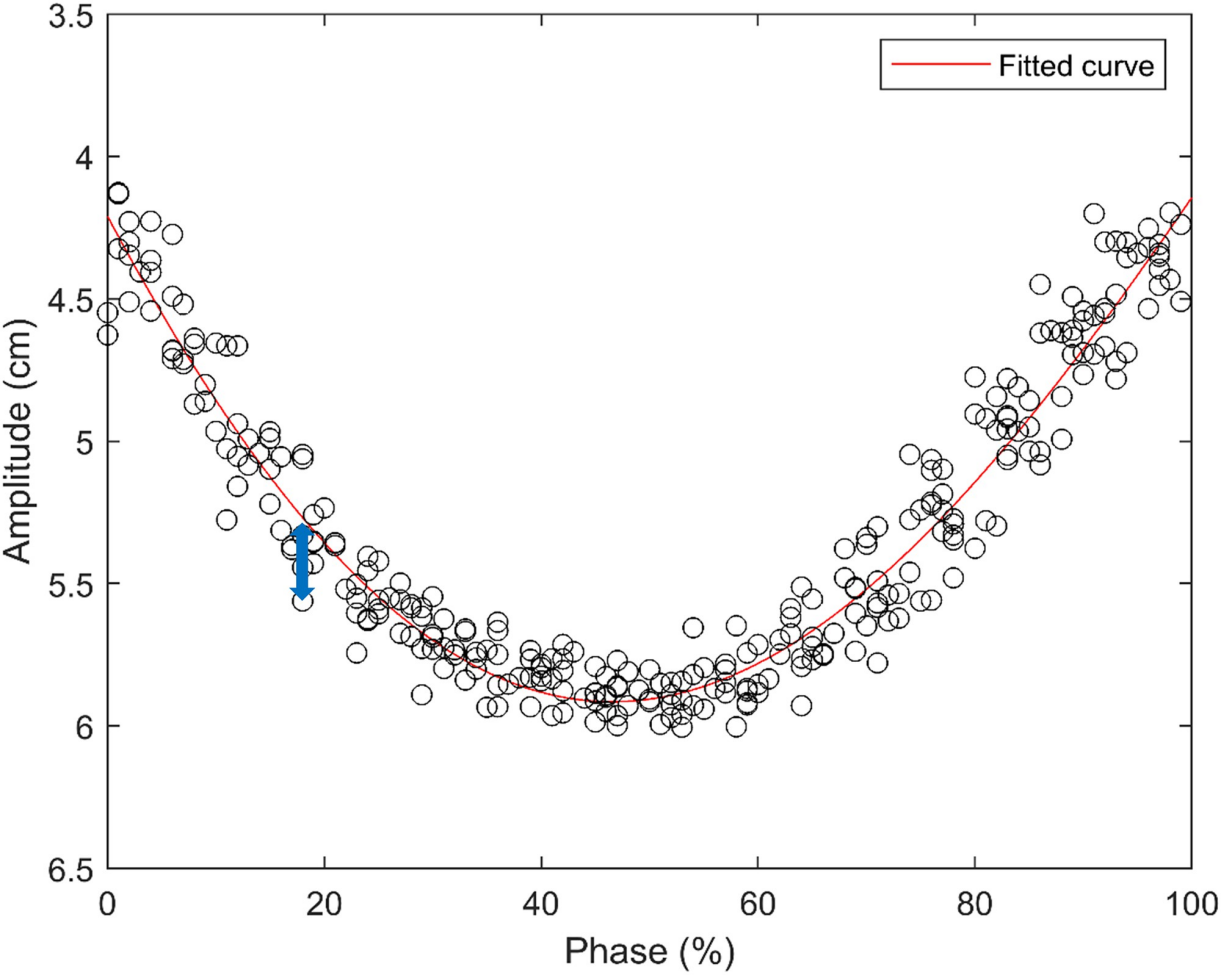
Plot of amplitude versus phase information of the surrogate respiratory signal and the representative respiratory signal fitted to the combination of sinusoidal functions.

*2*.*2*.*2*.*1*. *Modelling of a representative respiratory signal*. A procedure for modelling a representative respiratory signal from the RPM signal is conducted to establish a reference of respiratory amplitude information. Fig 6 shows an example of the surrogate respiratory signal obtained using the RPM system during the 4DCT scan, with blue circles indicating CT acquisition points. Amplitude information of CT acquisition points can be plotted against phase, as displayed in Fig 7. Curve-fitting can be performed, as marked in the red line, on the cluster of those CT acquisition points to determine a curve-fitted result as the reference of the respiratory signal. In this work, the combination of sinusoidal functions was selected for curve-fitting, based on the assumption in many previous publications that the regular respiratory signal can be accurately represented by the combination of sinusoidal functions [37–44].

**Fig 8:**
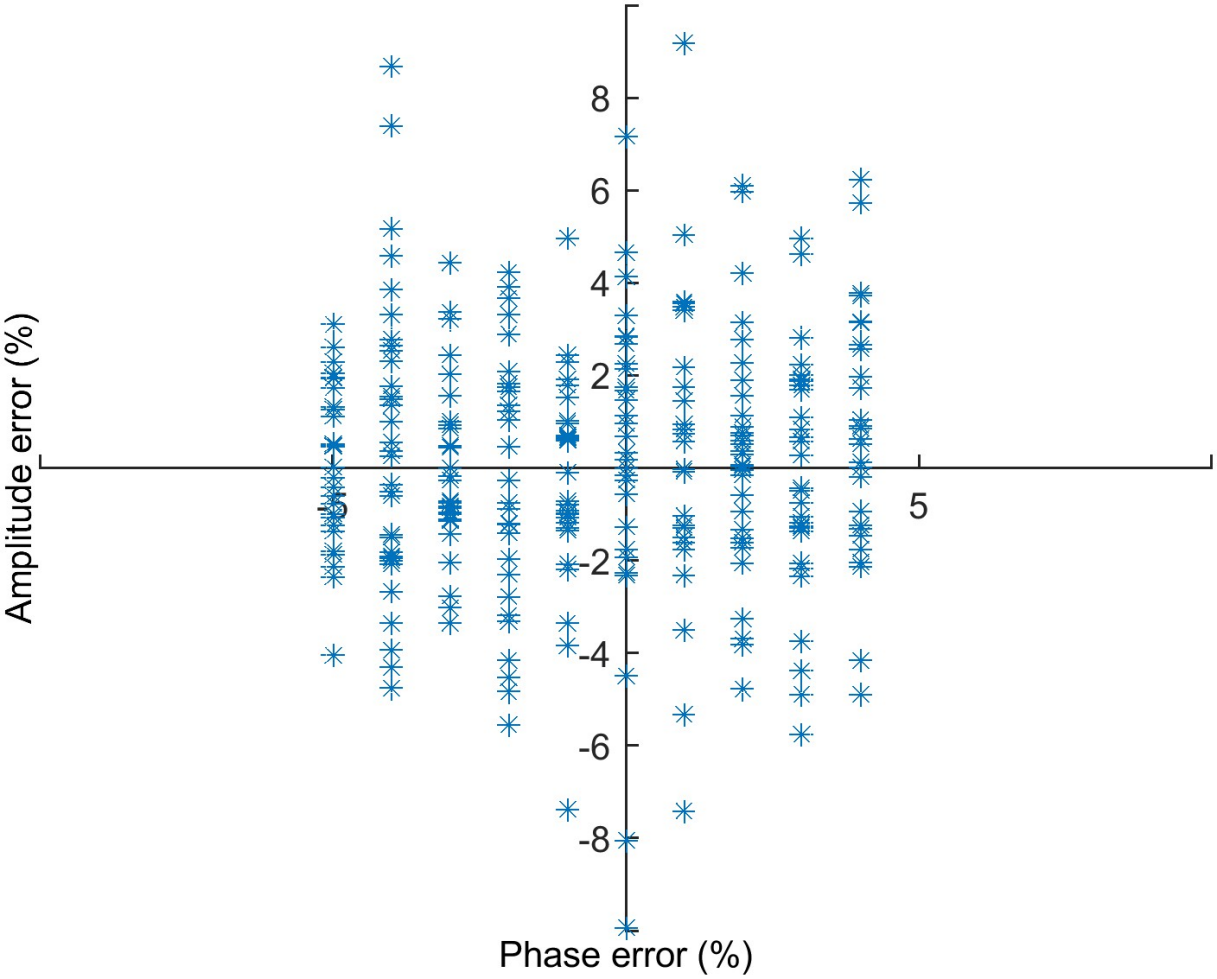
Scatter plot of phase and amplitude error for each CT acquisition point.

*2*.*2*.*2*.*2*. *Calculation of amplitude errors based on the representative respiratory signal*. Using the representative respiratory signal as the ground truth, the amplitude error at each CT acquisition point can be calculated. In this work, a normalisation procedure was applied to adjust patient-specific amplitude respiratory information to a uniform range. Fig 8 shows a scatter plot of phase and amplitude error for each CT acquisition point, demonstrating that the scale of phase and amplitude errors are similar so that it is possible to be employed in 4DCT sorting.

$$Amplitude\;error\left(x\right)=\frac{RPM\left(x\right)-REF\left(x\right)}{\text{max}\left(RPM\right)-\text{min}\left(RPM\right)}*100\left(\%\right),$$

, *where x = time at the CT acquisition point*,

RPM = surrogate respiratory signal,

REF = the representative respiratory signal

#### 2.2.3. Calculation of the quantitative indicator according to the selection of CT images

**Fig 9:**
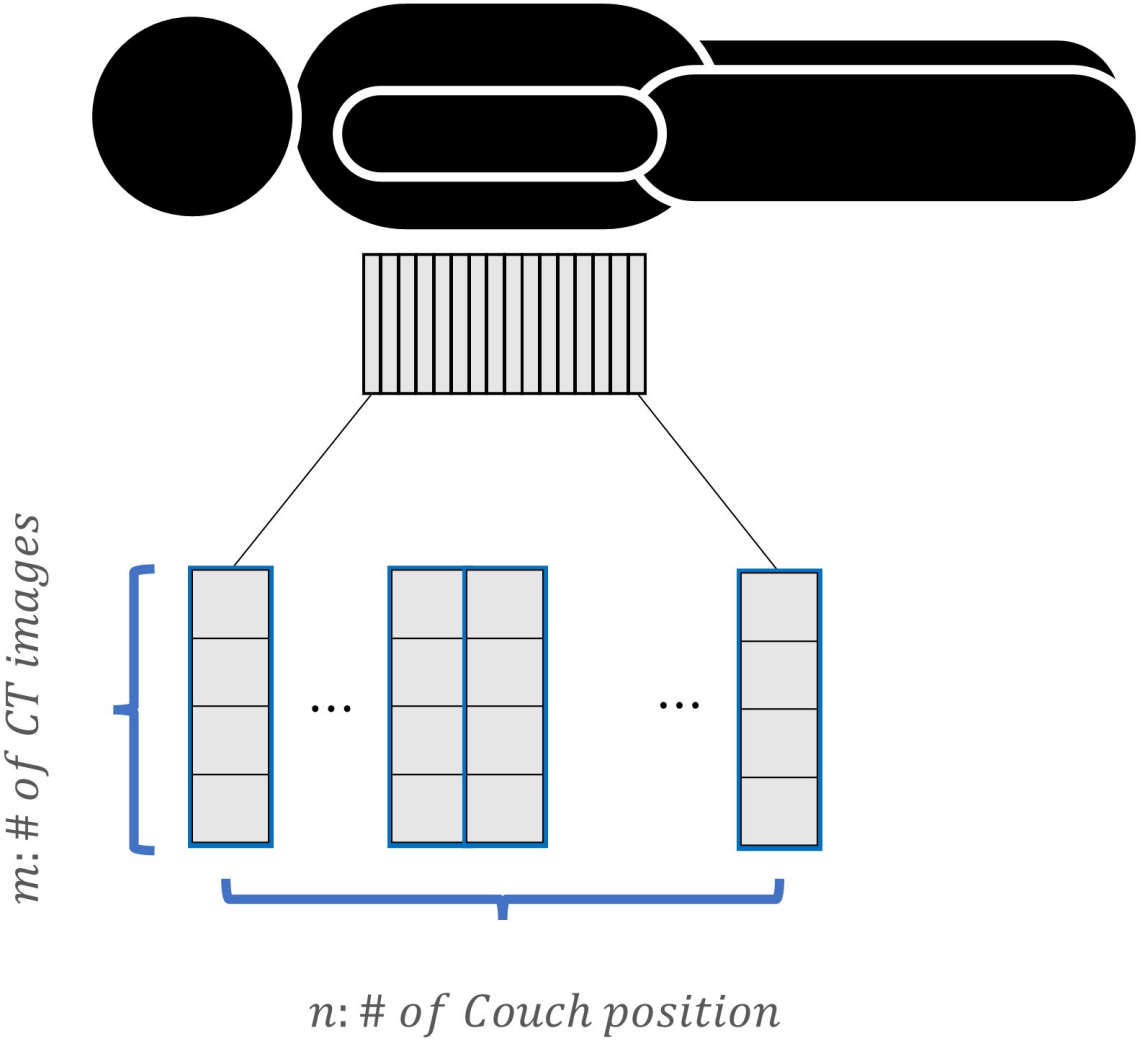
Schematic of 4DCT acquisition in cine mode.

Because the degree of RMA differs depending on the results of 4DCT sorting, the values of quantitative indicators should be checked for the number of cases in which the images are selected. If the number of CT images acquired from each couch position is *m* and the number of couch positions is *n*, as displayed in Fig 9, then the total number of cases to be calculated would be *m*^*n*^. However, as the quantitative indicator for 4DCT uncertainties inherently refers only to adjacent slices, the results would not be affected by other slices except the slices used for the calculation. Therefore, the effective number of cases that need to be calculated would be reduced to *m*^2^ × (*n* − 1). The indicators are calculated in advance for every case of image selection, and these values are tabulated as a look-up table.

#### 2.2.4. Selection of CT images considering all amplitude, phase, and RMA indices

**Fig 10:**
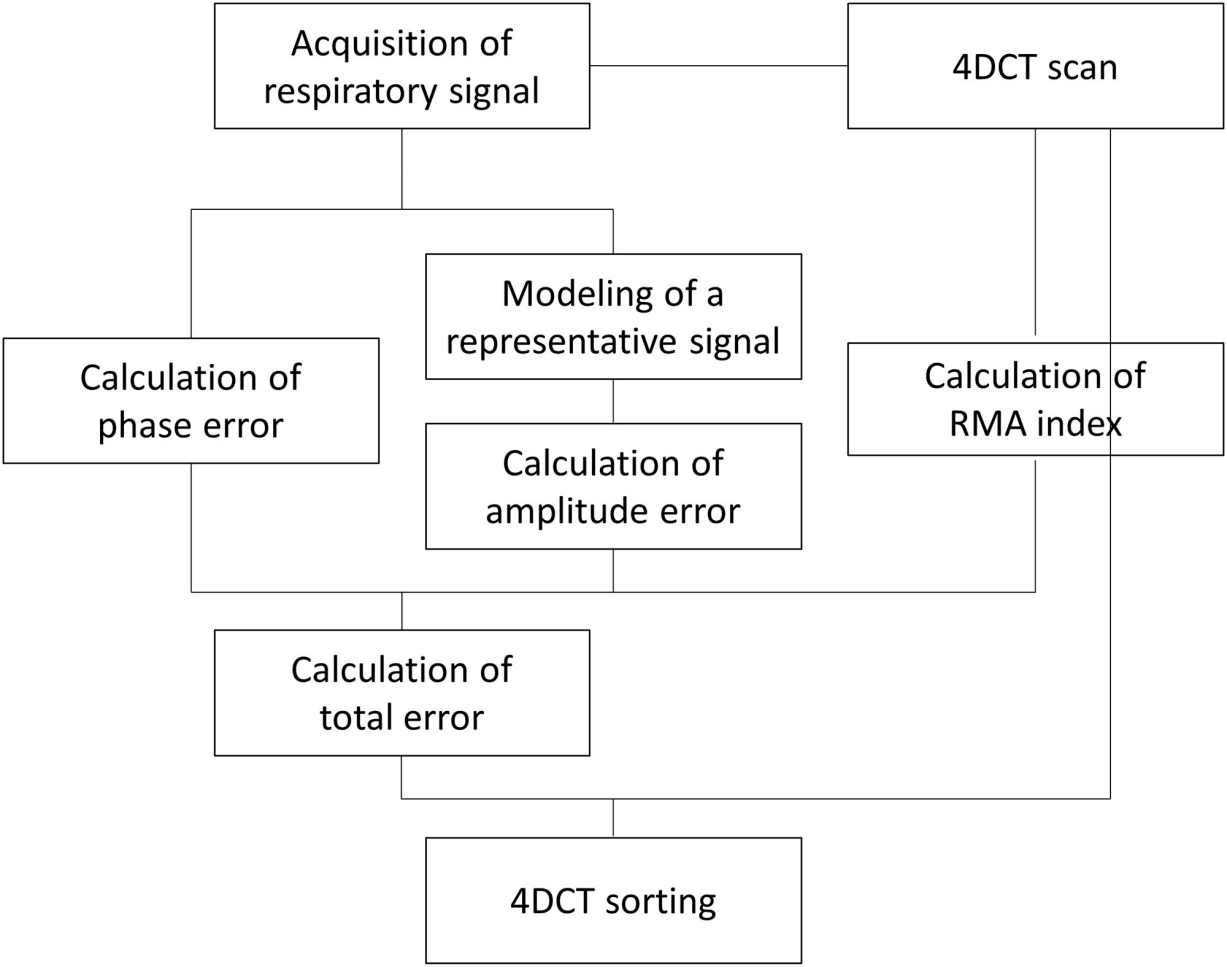
Conceptual workflow of the proposed amplitude-weighted phase sorting method with image similarity.

As mentioned in Method 2.1, Amp-sim-w algorithm that we proposed in this paper is a method in which amplitude error and RMA indices are added as the sorting criteria in the existing phase sorting framework. By accounting for all three types of information, the selected image set with the lowest cost function value is determined as the final result of 4DCT sorting. As described below, one can select CT images with the least total error, expressed as the root sum of squares of phase error, amplitude error, and RMA index. Since phase error, amplitude error, and RMA index are all normalised values ranging from 0 to 1, respectively, it can be employed by simply adding objective functions without significantly modifying the workflow of the existing phase sorting. In this study, the weighting factor for each of the three types of information was designated as 1/3 for each type. The overall workflow of the proposed amplitude-weighted phase sorting method with image similarity is summarised in Fig 10.


$$Total\;error:\sqrt{A\times Amplitude\;error^{2}+B\times Phase\;error^{2}+C\times RMA\;index^{2}},A,B,C:weighting\;factor\left(0<A,B,C<1,A,B,C=\frac{1}{3}\right)$$

### 2.3. Experimental conditions

To compare and evaluate the RMA indices and demonstrate the proposed 4DCT sorting method, two types of patient CT data, BHCT and 4DCT, were utilised. Forty patients who underwent radiotherapy for thoracic or abdominal cancers at our institute between May 2019 and August 2019 were included in this study. First, BHCT data were used to determine the extreme value of the RMA indicator for each candidate; 10 sets were used in this paper. Next, 4DCT data were used to check the correlation between operators’ visual criteria and the RMA metrics. Moreover, they were also employed to evaluate the performance of the proposed method. A total of 30 cases of 4DCT data were utilised in this study: 15 cases for the thoracic regions and 15 cases for the abdominal regions.

All BHCT and 4DCT in the experimental studies were acquired using a 16-slice CT scanner (GE Discovery CT590 RT, GE Healthcare, Milwaukee, Wisconsin, USA) with the RPM system (Varian Medical Systems, Palo Alto, California, USA). BHCT data were scanned in helical mode with the pitch as 0.938:1. 4DCT data were scanned in cine mode. The number of reconstructed breathing phases was set as 10. CT slice thickness was 2.5 mm, and 8 slices were acquired at each couch position. The time for a full gantry rotation was 0.5 seconds. The cine time interval, variable depending on the respiratory period, was usually set as 0.3 seconds. The cine acquisition duration per couch position, also variable according to the respiratory period, was usually set as 4 to 5 seconds to secure data for more than one breathing cycle. The aforementioned two parameters were determined after confirming the patient’s respiratory period through pre-scan respiratory education and monitoring. For 4DCT sorting, the GE Advantage 4D software was utilised to sort 4DCT images based on phase information acquired from the RPM system. As mentioned in the introduction, one acquires 4DCT images based on the conventional phase sorting, followed by an additional 4DCT set with manual sorting, if needed. CT images for each breathing phase level can be individually selected on the GE Advantage 4D software. Since the original sorting scheme of the software is set as phase sorting, CT images with the least phase error are automatically selected by default. If severe RMA is observed in the CT images even though 4DCT phase sorting is applied, then manual sorting is additionally performed. Based on the visual inspection, an experienced operator manually deselects the CT slices with significant RMA and replaces with the most probable alternative image within the similar respiratory phase level.

## 3. Results

### 3.1. Comparison of quantitative indices for RMA

#### 3.1.1. Evaluation of the extreme value of quantitative indices

The calculated values of each quantitative index for the thoracic and abdominal regions from 10 BHCT datasets were tabulated in Table 2. The numerical values of quantitative indicators, without the additional normalisation procedure including NCC and RMSD, were significantly different from the ideal maximum or minimum value despite the 4DCT uncertainty-free data. The results showed that those metrics without additional normalisation employed in previous researches so far have poor reliability for RMA indication. Moreover, it can be seen that there are considerable deviations depending on the body parts. Taking RMSD as an example, the averaged value in thoracic regions was 0.693. However, the value in abdominal regions was 0.380. However, DCC showed nearly maximum value in 4DCT uncertainty-free data regardless of the body part.

**Table 2:** The averaged value of the quantitative indicators for thoracic and abdominal regions from BHCT data.

		Body parts	
		Thorax	Abdomen
w/o normalisation	NCC	0.954±0.007	0.925±0.017
	RMSD	0.693±0.052	0.380±0.039
	DCC	0.995±0.003	0.998±0.002
w/ normalisation	N-NCC	0.999±0.002	0.999±0.008
	N-RMSD	0.013±0.006	0.031±0.028
	N-DCC	1.000±0.003	1.000±0.002

In summary, NCC and RMSD, except DCC, could not represent the maximum or minimum value in 4DCT uncertainty-free data and had different sensitivities depending on the body part. In contrast, N-NCC, N-RMSD, and N-DCC, which underwent additional normalisation procedure, showed values close to 1 or 0 regardless of the body part. Thus, it was confirmed that DCC and the metrics with the additional normalisation process are much consistent quantitative indicators for 4DCT sorting.

#### 3.1.2. Correlation of the quantitative index with the operator’s qualitative standard

**Fig 11:**
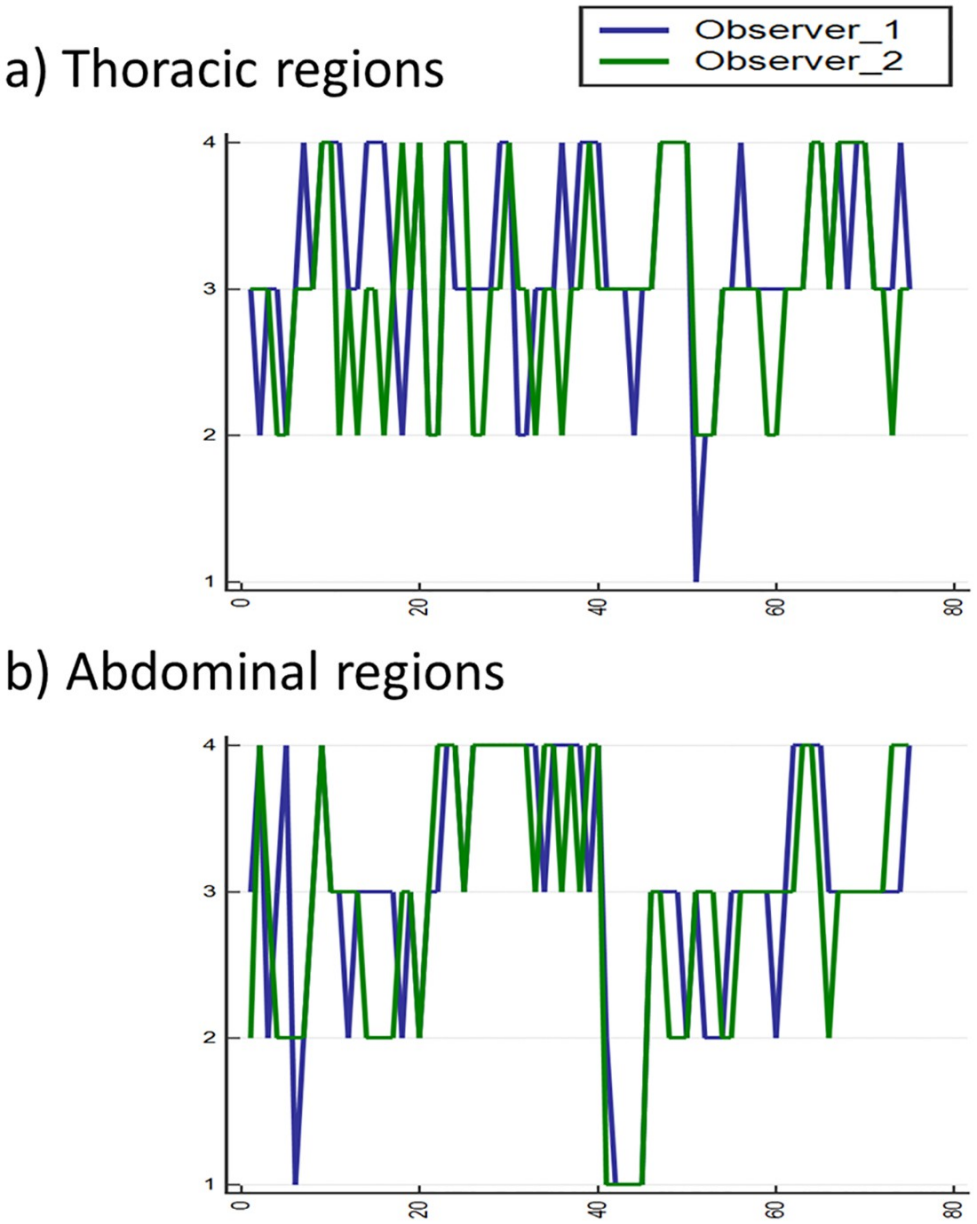
Scoring results for 30 patients (150 slices) evaluated by two operators performing manual sorting of 4DCT. a) Thoracic regions of 15 patients (75 slices) b) Abdominal regions of 15 patients (75 slices).

We also investigated the correlation between the RMA indicators and visual criteria of the operators performing manual sorting of 4DCT. First, to quantify the visual criteria of two operators, the degree of 4DCT uncertainties of five sections near the isocentre was evaluated. Thirty cases of phase sorted 4DCT, equivalent to 150 slices, consisting of 75 thoracic and 75 abdominal regions, were scored from 1 to 4 points, as displayed in Fig 11. After quantifying the visual standards, a weighted kappa analysis was performed to evaluate the inter-rate agreement of the score given by two operators. The statistical analysis was conducted using IBM SPSS Statistics (IBM Co., Armonk, NY, USA). The weighted kappa coefficient values of the thoracic and the abdominal regions of 4DCT were 0.47 and 0.71, respectively, with an average value of 0.62. Referring to Table 3, which distinguishes the agreement strength according to the weighted kappa coefficient values [45], it can be interpreted that the visual evaluation criteria of the two workers were consistent with a level ranging from ’moderate’ to ’good’. To sum up, with reliable inter-observer consistency, qualitative criteria for RMA were well quantified; thus, it was confirmed that these scores could be employed to evaluate RMA indicators.

**Table 3:** Agreement strength according to the weighted kappa coefficient values.

Weighted kappa coefficient	Agreement strength
< 0.20	Poor
0.20–0.40	Fair
0.41–0.60	Moderate
0.61–0.80	Good
0.81–1.00	Very Good

After converting the operator’s qualitative standards to uncertainty scores, the quantitative indicators are calculated in each evaluation region of phase-sorted 4DCT. Then through Pearson correlation analysis, the correlation between the quantitative index and the averaged uncertainty scores of two operators was evaluated, as shown in Table 4. Referring to Tables 4 and 5, it can be seen that the quantitative indicators with additional normalisation procedures have higher absolute values of *R* in both thoracic and abdominal areas than those without normalisation processes. N-RMSD showed the highest correlation coefficient value within a group of the quantitative indices. In summary, the quantitative indicators with additional normalisation processes have more consistent values than the other metrics for 4DCT sorting. N-RMSD has the highest correlation with the visual criteria of workers who perform manual sorting of 4DCT. Thus, from the result of quantitative index studies, N-RMSD was selected for the proposed Amp-sim-w method.

**Table 4:** Results of Pearson correlation analysis (R) for the RMA index and the visual standard of the operators who perform manual 4DCT sorting.

Index	Thorax		Abdomen	
	R	*p*	R	*p*
NCC	-0.220	0.058	-0.303	<0.01
DCC	-0.161	0.168	-0.348	<0.01
RMSD	0.169	0.147	0.566	<0.01
N-NCC	-0.506	<0.01	-0.471	<0.01
N-DCC	-0.364	<0.01	-0.310	<0.01
N-RMSD	0.625	<0.01	0.673	<0.01

**Table 5:** Interpretation of the Pearson correlation coefficient values.

R	Interpretation
+0.7 ~ +1.0 (-0.7 ~ -1.0)	High positive (negative)
+0.3 ~ +0.7 (-0.3 ~ -0.7)	Moderate positive (negative)
+0.1 ~ +0.3 (-0.1 ~ -0.3)	Low positive (negative)
+0.0 ~ +0.1 (+0.0 ~ -0.1)	Negligible

### 3.2. Performance evaluation of the proposed method

**Fig 12:**
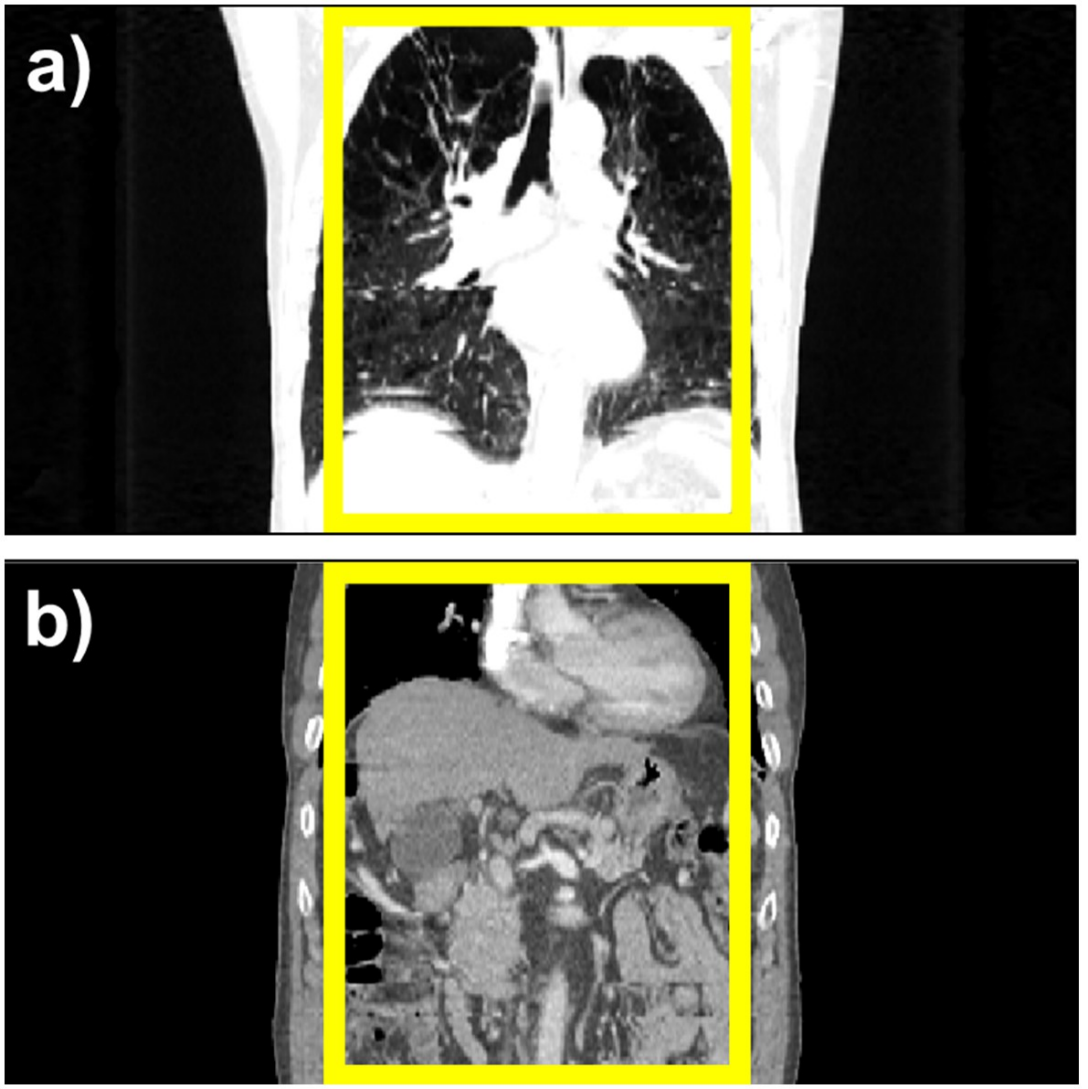
Coronal view of sorted 4DCT and its evaluation regions for performance comparison of the sorting algorithm.

**Fig 13:**
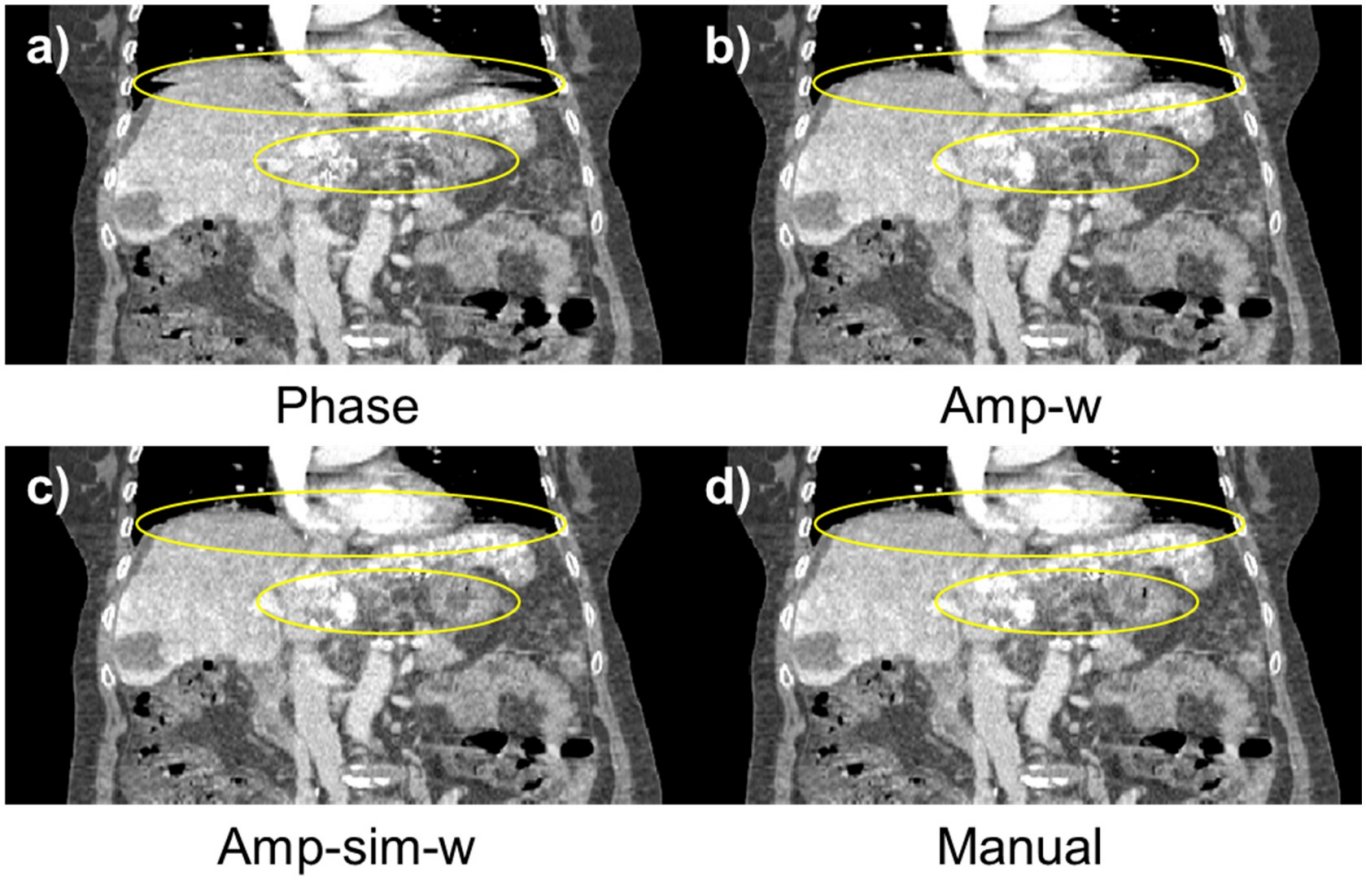
Coronal view of sorted 4DCT of the abdominal region according to the sorting algorithm. (Patient #33) a) conventional phase sorting, b) amplitude-weighted phase sorting, c) amplitude-weighted phase sorting with image similarity, and d) manual sorting method.

**Fig 14:**
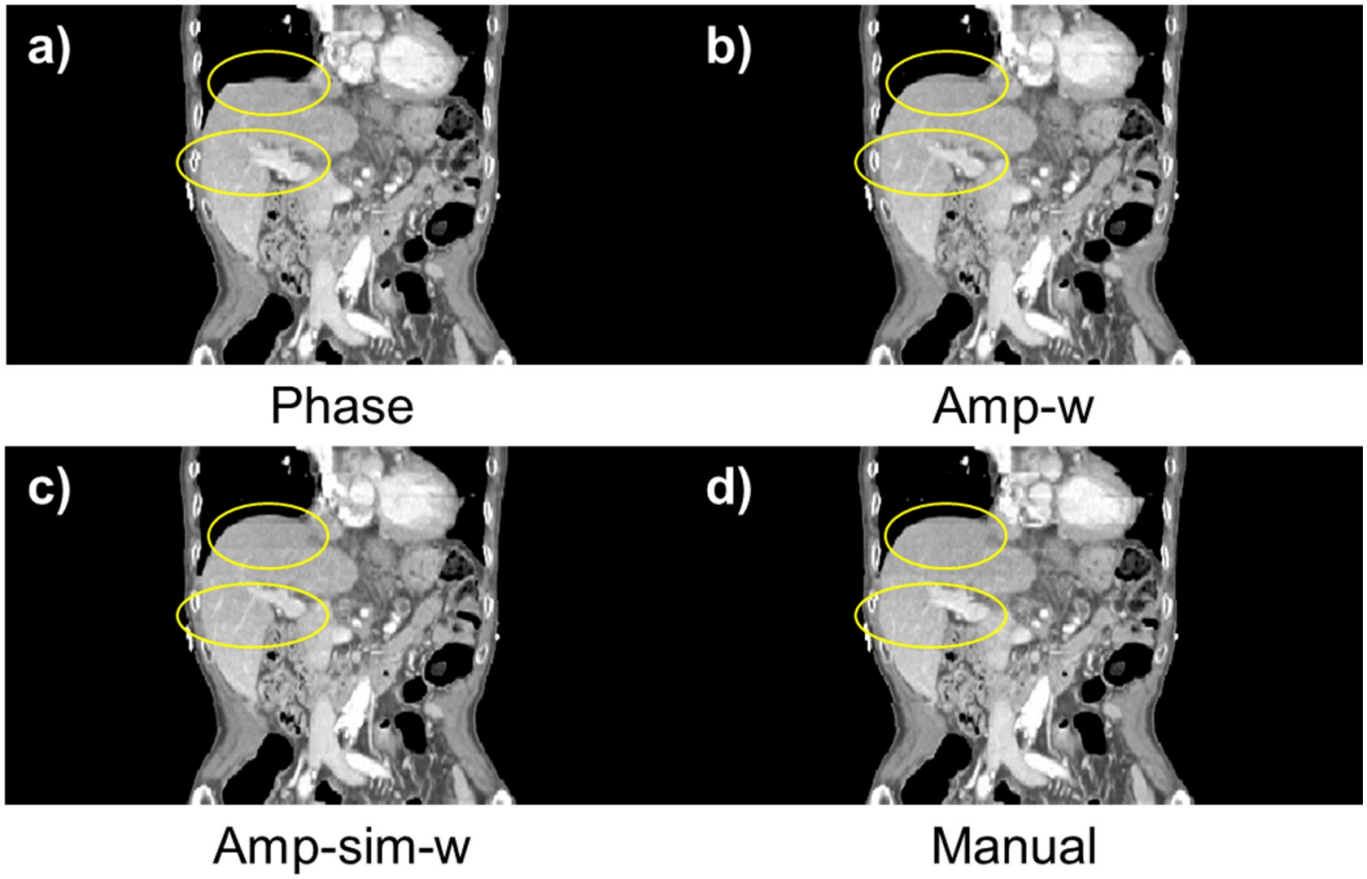
Coronal view of sorted 4DCT of the abdominal region according to the sorting algorithm. (Patient #1) (a) conventional phase sorting, b) amplitude-weighted phase sorting, c) amplitude-weighted phase sorting with image similarity, and d) manual sorting method.

**Fig 15:**
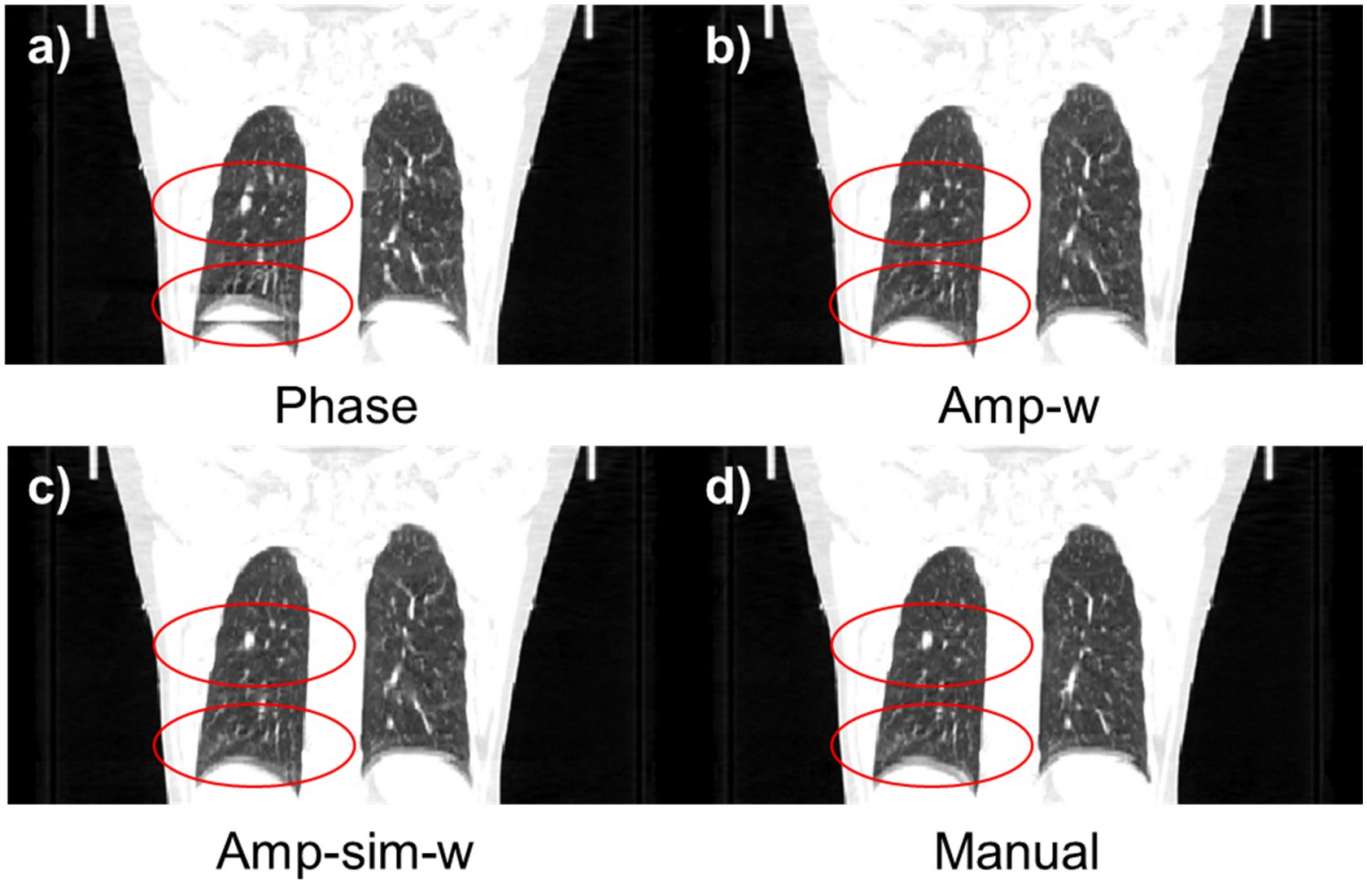
Coronal view of sorted 4DCT of the thoracic region according to the sorting algorithm. (Patient #19) (a) conventional phase sorting, b) amplitude-weighted phase sorting, c) amplitude-weighted phase sorting with image similarity, and d) manual sorting method.

**Fig 16:**
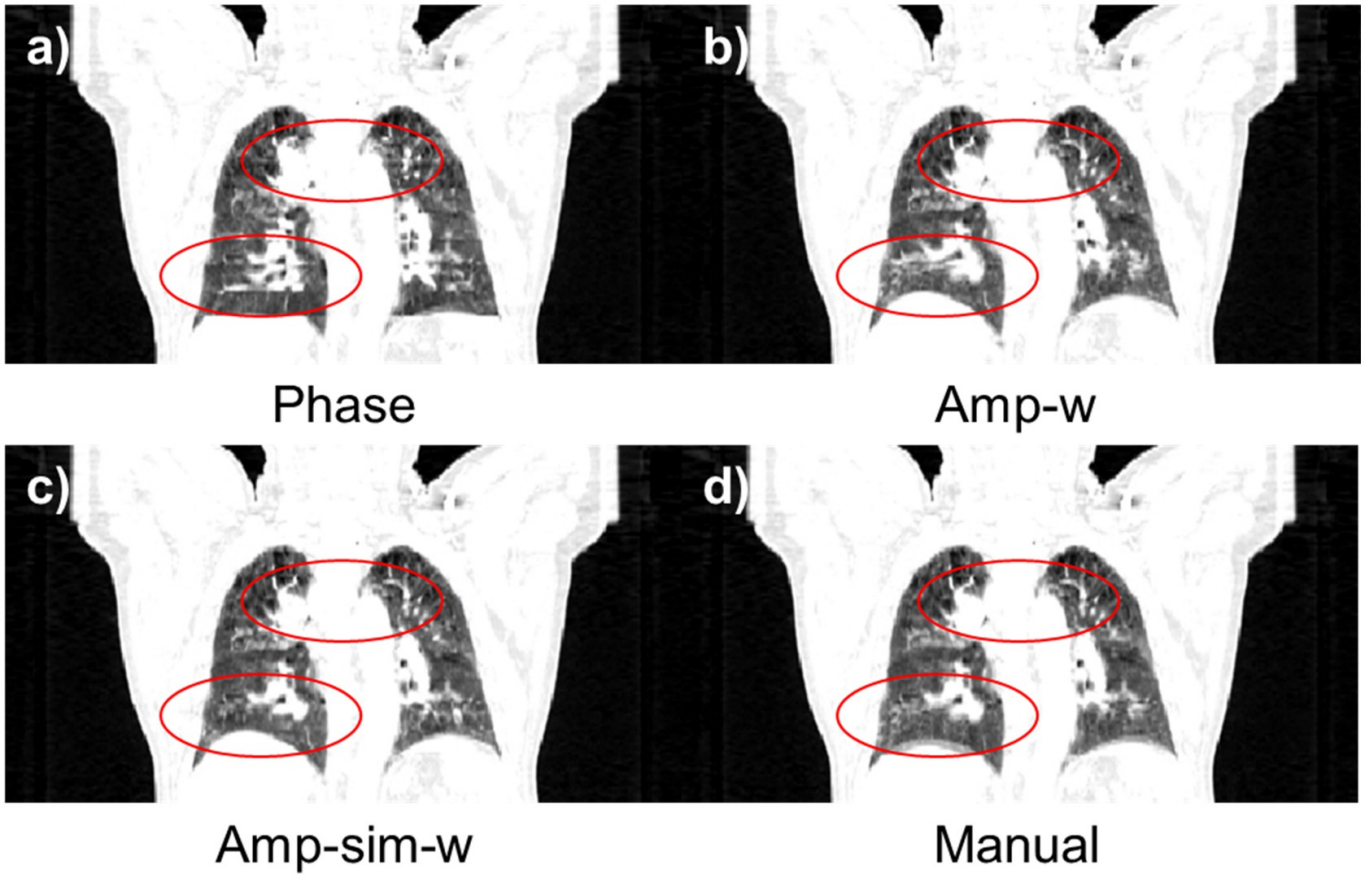
Coronal view of sorted 4DCT of the thoracic region according to the sorting algorithm. (Patient #16) (a) conventional phase sorting, b) amplitude-weighted phase sorting, c) amplitude-weighted phase sorting with image similarity, and d) manual sorting method.

The performance of the proposed method, Amp-sim-w, was evaluated in comparison to the phase sorting and manual sorting method. Furthermore, Amplitude-weighted phase sorting method (Amp-w) which exclude RMA index, was also involved to validate the effect of the indicator. Since N-RMSD was employed in the proposed method, it may be unfair to evaluate the quality of resultant sorted images with the same quantitative index. Thus, together with N-RMSD, N-NCC was also utilised to evaluate the performance of those sorting methods. As highlighted in the solid yellow box in Fig 12, the region of interests (ROI) was determined with a total width of 200 pixels for the quantitative evaluation, excluding the air region. Table 6a) and 6b) summarise the averaged values and those standard deviations of quantitative indicators according to the sorting algorithms. In the evaluation conducted with N-RMSD showing lower values at fewer RMA, the averaged value of the entire data was 0.3882 with phase sorting. However, the averaged values of the Amp-w and Amp-sim-w methods were 0.3189 and 0.3153, respectively, and it can be seen that those were comparable to 0.3237, which is the value with using the manual sorting method. Even when the results were analysed by dividing each body part, it was found that the proposed method is comparable to the manual sorting method for both thoracic and abdominal regions. Likewise, in the evaluation performed by N-NCC indicating higher values for fewer RMA, the proposed method showed similar performance to the manual sorting method. In summary, by using the proposed methods, both amp-w and amp-sim-w, RMA were successfully reduced better than when only phase sorting is applied. The performances were comparable to that of the manual sorting. According to the application of different sorting methods, the sample results of 4DCT sorting for each body part are displayed in Figs 13 and 14 for abdominal regions and Figs 15 and 16 for thoracic regions. As can be seen from the marked ROI, due to irregular respiration, 4DCT uncertainties still appeared in Figs 13–15 and 16(a) even though the phase sorting method was applied. However, by applying the proposed methods, the results were comparable to that of the manual sorting method, as shown in Figs 13–15, and 16(b)–16(d). In the case of Fig 16, 4DCT uncertainties still existed even though the Amp-w method was applied; it is presumed that the correlation between the surrogate signal and the internal motion is low. On the other hand, in the result of applying the Amp-sim-w method, those 4DCT uncertainties were also reduced.

**Table 6:** Mean and standard deviation of a) N-NCC and b) N-RMSD, respectively, in the sorted 4DCT according to the sorting algorithm.

N-NCC		Phase	Amp-w	Amp-sim-w	Manual
Total	Mean	0.9388	0.9496	0.9500	0.9486
	Std	0.0249	0.0234	0.0230	0.0224
Thorax	Mean	0.9355	0.9421	0.9429	0.9430
	Std	0.0228	0.0223	0.0215	0.0221
Abdomen	Mean	0.9421	0.9571	0.9571	0.9541
	Std	0.0272	0.0227	0.0229	0.0220
N-RMSD		Phase	Amp-w	Amp-sim-w	Manual
Total	Mean	0.3882	0.3189	0.3153	0.3237
	Std	0.1033	0.0799	0.0732	0.0699
Thorax	Mean	0.3772	0.3313	0.3260	0.3225
	Std	0.0754	0.0893	0.0786	0.0631
Abdomen	Mean	0.3992	0.3064	0.3046	0.3249
	Std	0.1272	0.0700	0.0685	0.0783

## 4. Discussion

We conducted experimental studies on quantitative metrics to indicate the degree of 4DCT uncertainties. From the results, we proposed an improved sorting algorithm for 4DCT and evaluated its performance compared to the existing methods, including conventional phase sorting and manual sorting methods. The essence of this study is to systematically evaluate the RMA indicators by using both BHCT and 4DCT with visual-criteria-based manual sorting and use the most suitable indices to improve the conventional phase sorting scheme.

In addition to the main purpose of this study mentioned above, it was possible to validate the overall performance of manual sorting currently being conducted in our institute. Although it is a subjective task performed by workers, inter-observer variability was within certain range, from ’moderate’ to ’good’. Also, it was confirmed that manual sorting has a reasonable correlation level with quantitative indicators and shows consistent 4DCT sorting results.

One of the limitations in this study is that the manual sorting method, which was considered as the ground truth for 4DCT sorting in this work, may be inadequate as a gold standard. Because manual sorting is a nonquantitative task that mainly relies on the visual criteria of workers, it may have intervariability between workers, which may result in inconsistent results. Human errors are another factor leading to suboptimal results of manual sorting, as shown in Fig 14d). Thus, paradoxically, the limitations of the manual sorting method mentioned above can highlight the advantages of the proposed sorting method, which have quantitative power and consistency.

In most cases which are presumed to high correlation between the internal organ movement and the surrogate respiratory signal, RMA were significantly reduced by introducing only amplitude information to the existing phase sorting method. However, in some cases, RMA were successfully removed after further introducing the quantitative index as a criterion for 4DCT sorting. Overall, the proposed sorting method using both amplitude information and the quantitative indicator showed the best performance in terms of RMA reduction. However, the use of CT data causes an additional calculation burden. Therefore, further studies on the improved framework for data transmission and processing would be required before clinical implementation. Thus, depending on the 4DCT workflow of their sites, sufficient considerations by medical doctors and physicists would be necessary to apply the proposed method.

**Fig 17:**
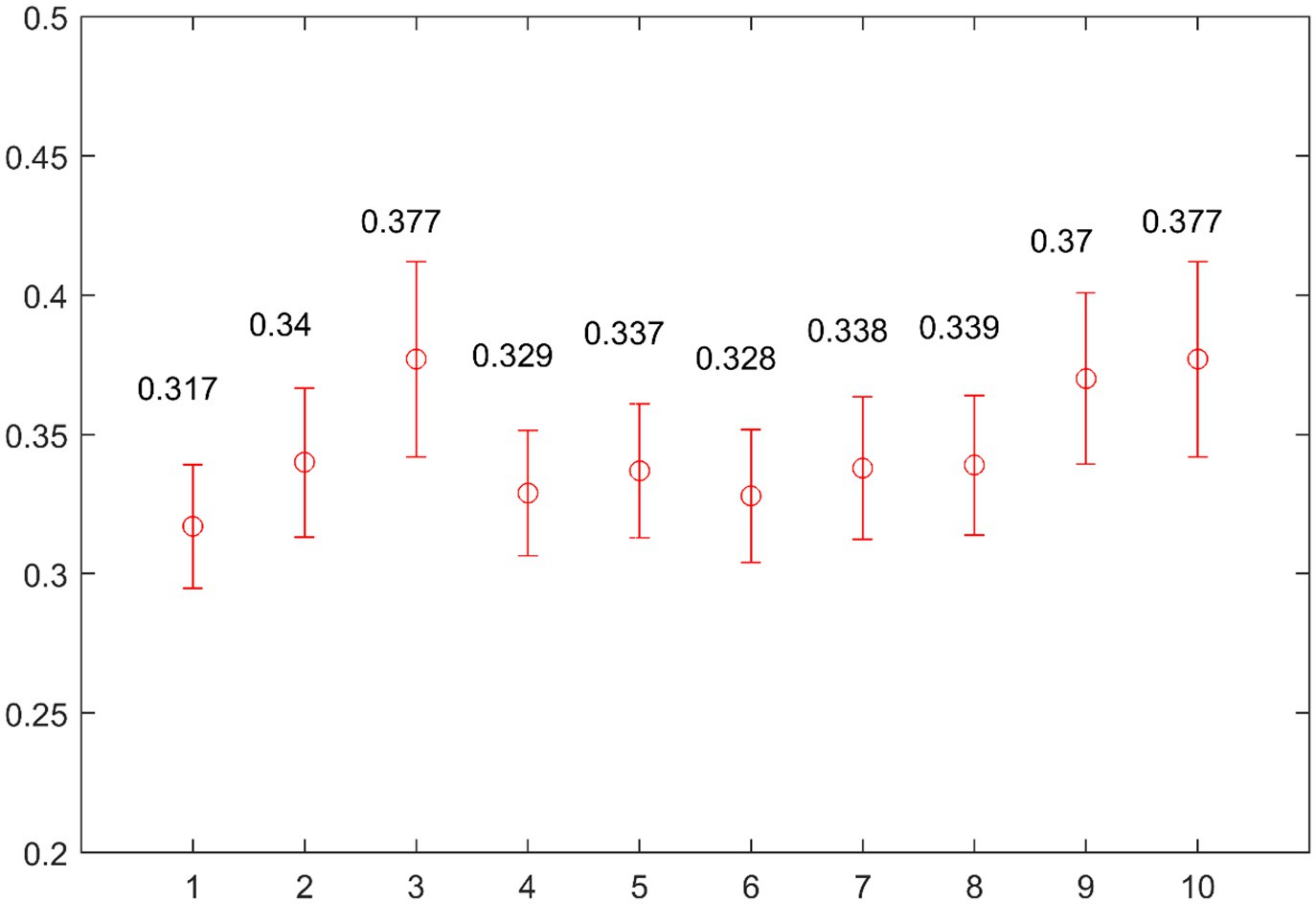
Mean and standard deviation of N-RMSD in the 4DCT sorted by proposed Amp-sim-w method according to the ratio of each objective information.

For more robust and consistent 4DCT sorting algorithms, further research for an optimisation process that determines the weighting factors of each objective information would be required. Depending on the weighting factors, the resultant images of 4DCT sorting may vary slightly. The respiratory condition of the patients and the position of the body where 4DCT were scanned would be the main factors that determine the appropriate weighting factors. To find the optimal weighting factors in this study, we adjusted the weighting factors of phase information, amplitude information, and the quantitative indicator as summarised in Table 7. We evaluated the sorting results for each circumstance. Among the weighting conditions used in the 30 cases of 4DCT, the ratio of 1:1:1 showed the lowest mean and standard deviation values of N-RMSD, as shown in Fig 17. Thus, the ratio of 1:1:1 was employed in this study.

**Table 7:** Ratio values for the configuration of each objective information for 4DCT sorting in the proposed Amp-sim-w method.

	Weighting ratio		
	Phase	Amplitude	RMA indicator (N-RMSD)
1	1	1	1
2	0	1	1
3	1	0	1
4	1	1	2
5	2	1	1
6	1	2	1
7	0	1	2
8	0	2	1
9	1	0	2
10	2	0	1

In future works, the proposed 4DCT sorting method would be applied to many diverse clinical cases, together with in-depth evaluation. On top of the direct search to find the best result of image sorting exploited in this study, we also plan to use various types of optimisation algorithms to find the global optimum efficiently. The overall calculation time for checking the number of all cases for one patient in this work was approximately 10 minutes using CPU-based computing. However, we believe that it could be accelerated by employing optimisation algorithms and a GPU-based environment. Moreover, by using the RMA index explored in this study, we also envision that one can provide a guideline of manual sorting to operators, which is also being planned as a further study.

## 5. Conclusion

In this study, we conducted experimental studies on quantitative metrics to indicate the degree of 4DCT uncertainties generated by the irregular respiration of the patient during a 4DCT scan. Based on the results, we proposed improved sorting algorithms for 4DCT and evaluated their performance compared to existing algorithms. The results of those studies showed that RMA can be effectively reduced compared to the current phase sorting algorithm without significant modification of the conventional 4DCT workflow.

## Supporting information

S1 Data (XLSX)

S2 Data (XLSX)

S3 Data (XLSX)
